# Extrinsic post burn peri-anal contracture leading to sub acute intestinal obstruction: A case report

**DOI:** 10.1186/1757-1626-1-117

**Published:** 2008-08-21

**Authors:** Jagdeep S Thakur, CGS Chauhan, Vijay K Diwana, Anamika Thakur

**Affiliations:** 1 Department of ENT – Head & Neck Surgery, I G Medical College, Shimla, HP, 171001, India; 2 Dept of Plastic and Reconstructive Surgery, I G Medical College, Shimla, HP, 171001, India; 3 Dept of Pharmacology, I G Medical College, Shimla, HP, 171001, India

## Abstract

Peri-anal contracture lead to intestinal obstruction whenever there is involvement of anal orifice. In this case anus and peri-anal skin up to two cm was normal; however both gluteal folds were fused because of post burn scar leaving a very small opening which lead to faecal impaction and sub acute intestinal obstruction.

## Background

Management of the burn patient is the most challenging condition for the medical staff as the fate of the patient depends on the quality of the management provided during hospital stay and after discharge. Even if the patient recovers from the burn injuries, the development of the deformities overshadow the earlier management. This post burn reconstructive surgery and physiotherapy consultation needs to be made compulsory in the burn units.

## Case report

A two and half year old male child was admitted with complaint of progressive difficulty in passing stools along with progressive distension of abdomen, for last one year. There was history of vomiting, off and on for the last fortnight. Patient had history of sustaining 10% thermal burns over perineum, gluteal region and left foot about one and half year back.

**Figure 1:**
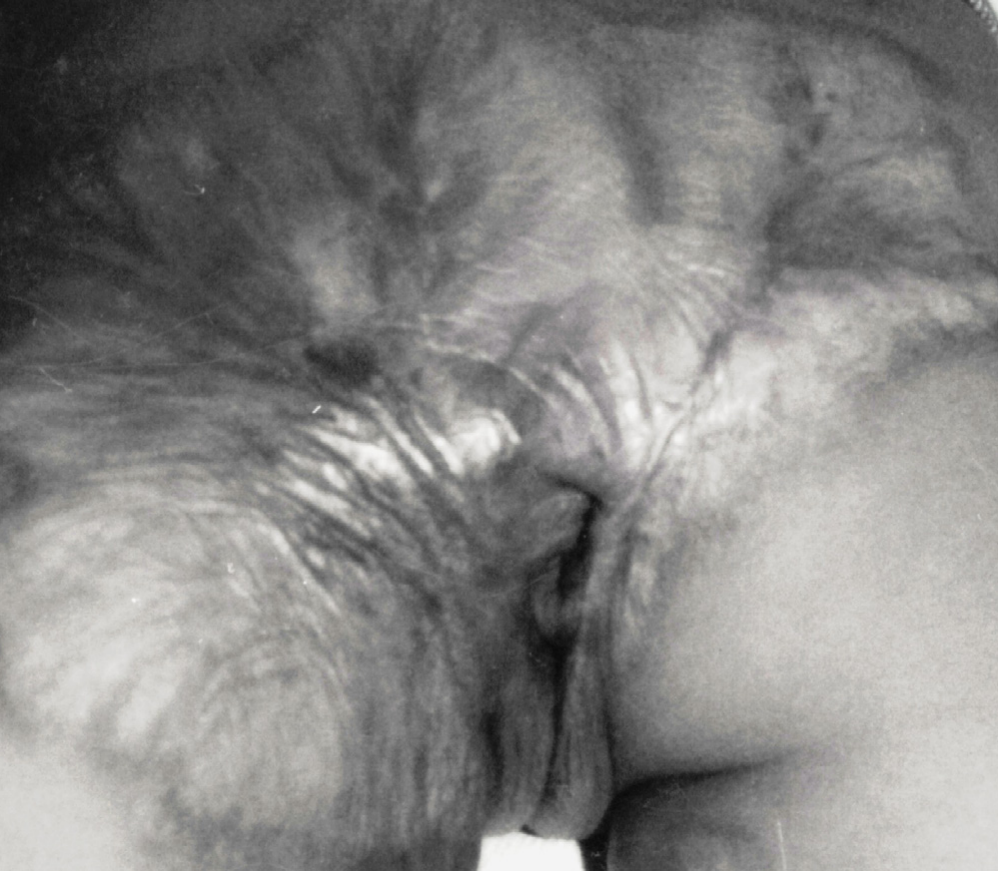
Peri-anal post burn contracture obstructing normal view of anal area.

On examination, there was mild distension of abdomen and occasional visible peristalsis movement with exaggerated bowel sounds. Examination of perineum showed that both the gluteal folds were fused because of post burn scar and there was a small opening approximately three mm in diameter in the centre (Figure 1). Rectal examination could not be carried out through this opening. The general physical and other systemic examination was normal.

**Figure 2:**
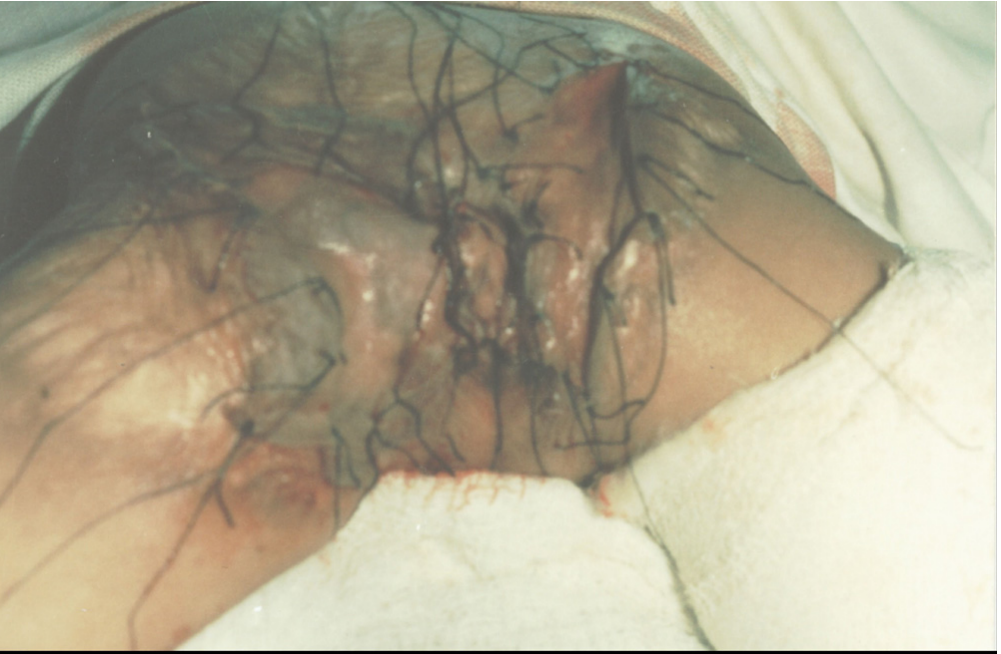
Intraoperative view with skin graft in place.

The blood investigations were normal and x-ray abdomen showed few air fluid levels. The child was operated under general anaesthesia. The contracture was released and both gluteal folds were separated. Raw area was grafted with split thickness skin graft. When contracture was released, it was found that anal verge along with peri-anal skin up to two cm was normal (Figure 2). It was fusion of gluteal folds due to post burn scar which led to sub acute intestinal obstruction. There was faecal impaction in the rectum. Post operative recovery was uneventful and graft was well taken.

## Discussion

**Figure 3:**
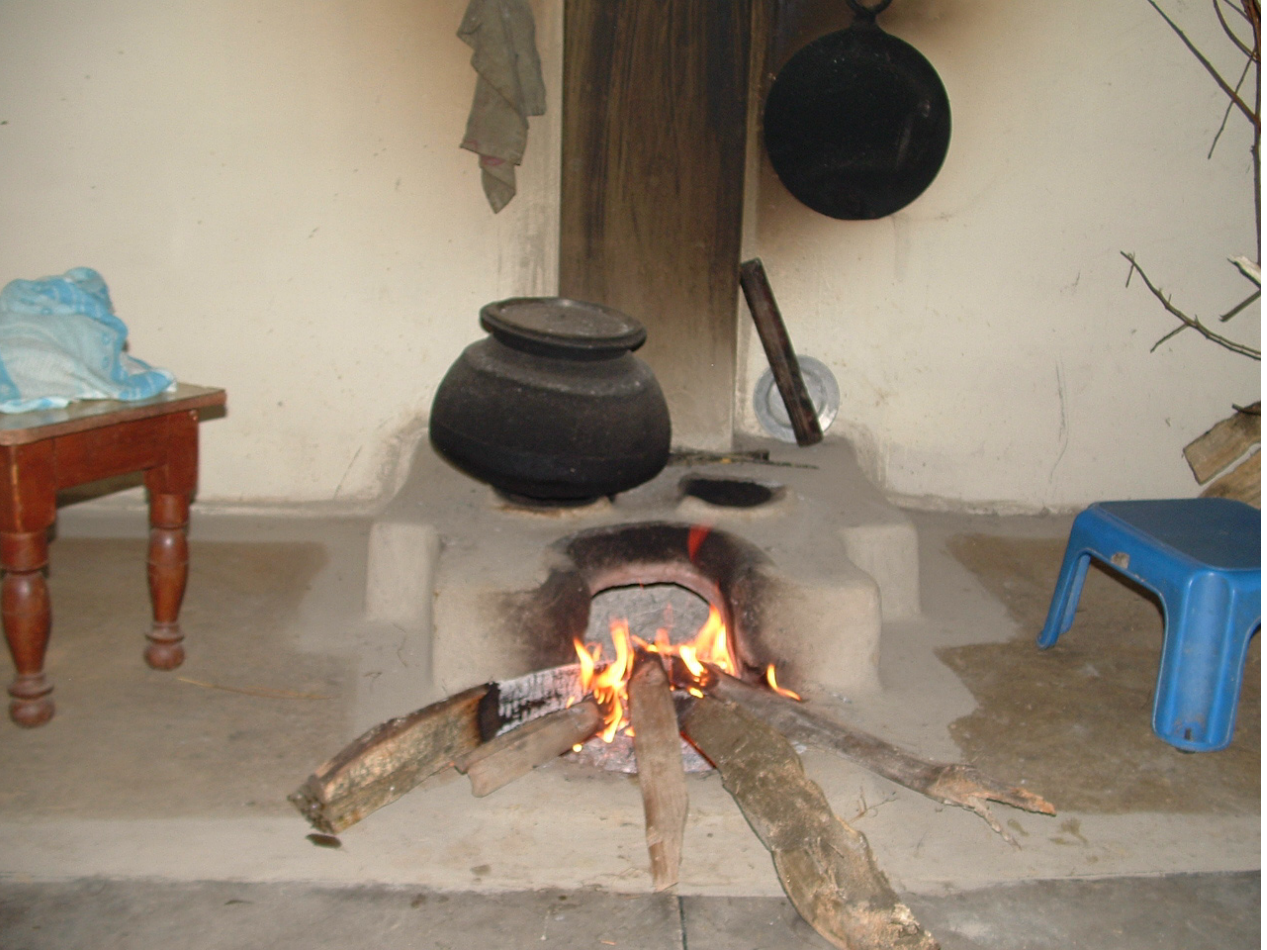
'**Chullah' a traditional stove**.

In the review of literature (Medline search), there are number of publications on the perineal burn and its management in the children [1-4]. As compared to this, we have found only one publication on perineal contracture leading to anal stricture and mega rectum in a three years old child [3]. In comparison to this report, in our case there was no involvement of anus or rectum. The intestinal obstruction was due to the post burn contracture in the gluteal fold which lead to the obstruction beyond the anus. This contracture was released and patient had complete recovery without any sequel. One more interesting fact in this case was that the patient sustained burn injury in gluteal area by sitting on the 'Chullah' (Figure 3), an earthen made stove, in which wood is used as fuel, a very common practice for the cooking in rural area of our country.

## Conclusion

Although perineal and gluteal burns are rare even in the rural areas of our country, as people are now using natural gases for the cooking etc but this rare case report emphasises on the critical burn care, post burn care, physiotherapy and regular follow up to the hospital E. Ye [4] has also given emphasises on the meticulous preoperative and post operative care in patients with chronic obstruction due to peri-anal contractures.

## Consent

The written informed consent of the patient has been obtained for the publication of this case report and accompanied images.

## Competing interests

The authors declare that they have no competing interests.

## Authors' contributions

JST has designed and written the article and is the principal contributor, CGS was involved with the management of the patient, conception, design and review of the article, VKD was involved in the management of the patient, conception and critical review of the article, AT was involved in acquisition of the data, review of the literature and critical review of the article. All the authors have read and given final approval for this article
